# Identification of Gene Modules Associated with Drought Response in Rice by Network-Based Analysis

**DOI:** 10.1371/journal.pone.0033748

**Published:** 2012-05-25

**Authors:** Lida Zhang, Shunwu Yu, Kaijing Zuo, Lijun Luo, Kexuan Tang

**Affiliations:** 1 Plant Biotechnology Research Center, School of Agriculture and Biology, Shanghai Jiao Tong University, Shanghai, China; 2 Shanghai Agrobiological Gene Center, Shanghai Academy of Agricultural Sciences, Shanghai, China; Inserm U869, France

## Abstract

Understanding the molecular mechanisms that underlie plant responses to drought stress is challenging due to the complex interplay of numerous different genes. Here, we used network-based gene clustering to uncover the relationships between drought-responsive genes from large microarray datasets. We identified 2,607 rice genes that showed significant changes in gene expression under drought stress; 1,392 genes were highly intercorrelated to form 15 gene modules. These drought-responsive gene modules are biologically plausible, with enrichments for genes in common functional categories, stress response changes, tissue-specific expression and transcription factor binding sites. We observed that a gene module (referred to as module 4) consisting of 134 genes was significantly associated with drought response in both drought-tolerant and drought-sensitive rice varieties. This module is enriched for genes involved in controlling the response of the plant to water and embryonic development, including a heat shock transcription factor as the key regulator in the expression of ABRE-containing genes. These results suggest that module 4 is highly conserved in the ABA-mediated drought response pathway in different rice varieties. Moreover, our study showed that many hub genes clustered in rice chromosomes had significant associations with QTLs for drought stress tolerance. The relationship between hub gene clusters and drought tolerance QTLs may provide a key to understand the genetic basis of drought tolerance in rice.

## Introduction

Drought is a major environmental stress factor that affects the growth and development of plants. Plant drought stress response is one of the most complex biological processes, and it involves numerous changes at the physiological, cellular, and molecular levels. The increasing molecular information has shown complex gene networks that consist of numerous different genes are involved in plant responses to drought stress [1], [2]. Many genes with various functions have been identified to be involved in the response of plants to drought stress, but little is known about the relationships between these genes. Thus, the current challenge for understanding the drought stress response is to discover the relationships between genes at a system-based level that leads to the formation of this complex process in plants.

The development of high-throughput data-collection techniques, such as microarrays and deep sequencing, enables gene function to be uncovered on a global scale. Microarray analyses can simultaneously measure the expression levels of a large number of genes, but may not provide much information on gene-gene interrelationships. Due to rapid advances in network biology, network-based analysis of large-scale datasets has offered a novel view of biology [3]. The gene co-expression network, which is one type of biological network, is constructed from microarray datasets that facilitate a global view of transcriptional relationships. This can help in the understanding of how genes interplay to carry out specific biological functions.

In recent years, global co-expression networks have been constructed for rice [4], [5] and other plants [6]–[9], which have provided an overview of gene-gene interrelationships at the system-based level. In addition, several online resources for plant gene co-expression networks, including CressExpress [10], ATTED-II [11] and RiceArrayNet [5], have been developed to enable the visualization and data mining of co-expression networks for biologists. Recent studies have shown that gene co-expression networks were also used to identify a set of candidate genes that are responsible for specific phenotypes in plants [12]–[14].

Rice (*Oryza sativa*), one of the most important cereal crops, has been used as a model plant for the grass family. A better understanding of complex interactions among drought-stress related genes is of great importance to improve the stress resistance of rice and other cereals. In this study, we used network-based gene clustering to identify drought-responsive modules to provide a better understanding of the underlying molecular mechanisms of drought response in rice. This method can help in uncovering gene interrelationships for studies into the complex response to drought stress in rice, as well as provide a reference for other crop plants.

## Results

### Identification of drought-responsive genes

A total of 815 Affymetrix rice arrays (Table S1) obtained from the NCBI Gene Expression Omnibus and EBI ArrayExpress Archive were used for network construction. In order to reduce network noise, only those genes whose expression levels can be distinguished by probe sets were used in the subsequent analysis. All probe sets were searched again with the MSU rice genome data (version 6.1) to map the probe sets to gene models; we identified 25,737 probe sets that had a one-to-one pattern of mapping to rice genes. The drought-responsive genes were identified based on the normalized expression profiling data of five experiment sets. Among the 25,737 unique one-to-one genes, a total of 2,607 genes were identified whose expression levels showed significant changes under drought stress conditions in two or more experimental sets.

### Construction of drought-responsive gene network

**Figure 1:**
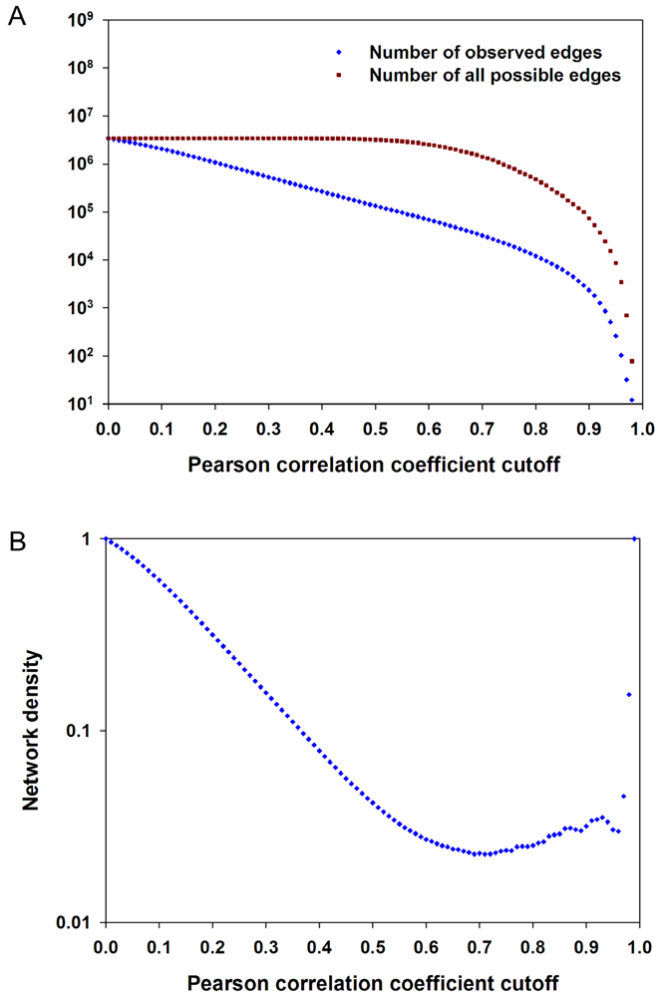
Choosing Pearson correlation coefficient cutoff value. A, the actual number of edges and all possible edges among the non-singleton nodes as a function of correlation coefficient cutoff values. B, network density at different correlation coefficient cutoff values.

The absolute Pearson correlation coefficients (*r*) were calculated for each pair of the 2,607 drought-responsive genes. To determine a biologically relevant correlation coefficient for gene co-expression, we examined the changes in the actual number of edges in the co-expression network and all possible edges in the control network consisting of these non-singleton nodes as a function of *r* cutoff values. As the cutoff increased, both the actual number of edges and all possible edges decreased; however, as the cutoff reached a relatively high value, the decreasing rate of the actual number of edges became slower than all possible edges (Figure 1A). This could lead to an increase in network density (a ratio of the actual number of edges to all possible edges among the non-singleton nodes). Indeed, as is shown in Figure 1B, the network density was at its minimum at the cutoff value of 0.71 and then increased as the cutoff value was greater than 0.71. The increase in network density was attributed to the presence of high *r*-value links that connected a decreasing number of nodes, indicating that biologically significant co-expressions are expected to be found above the *r*-value [15].

**Figure 2:**
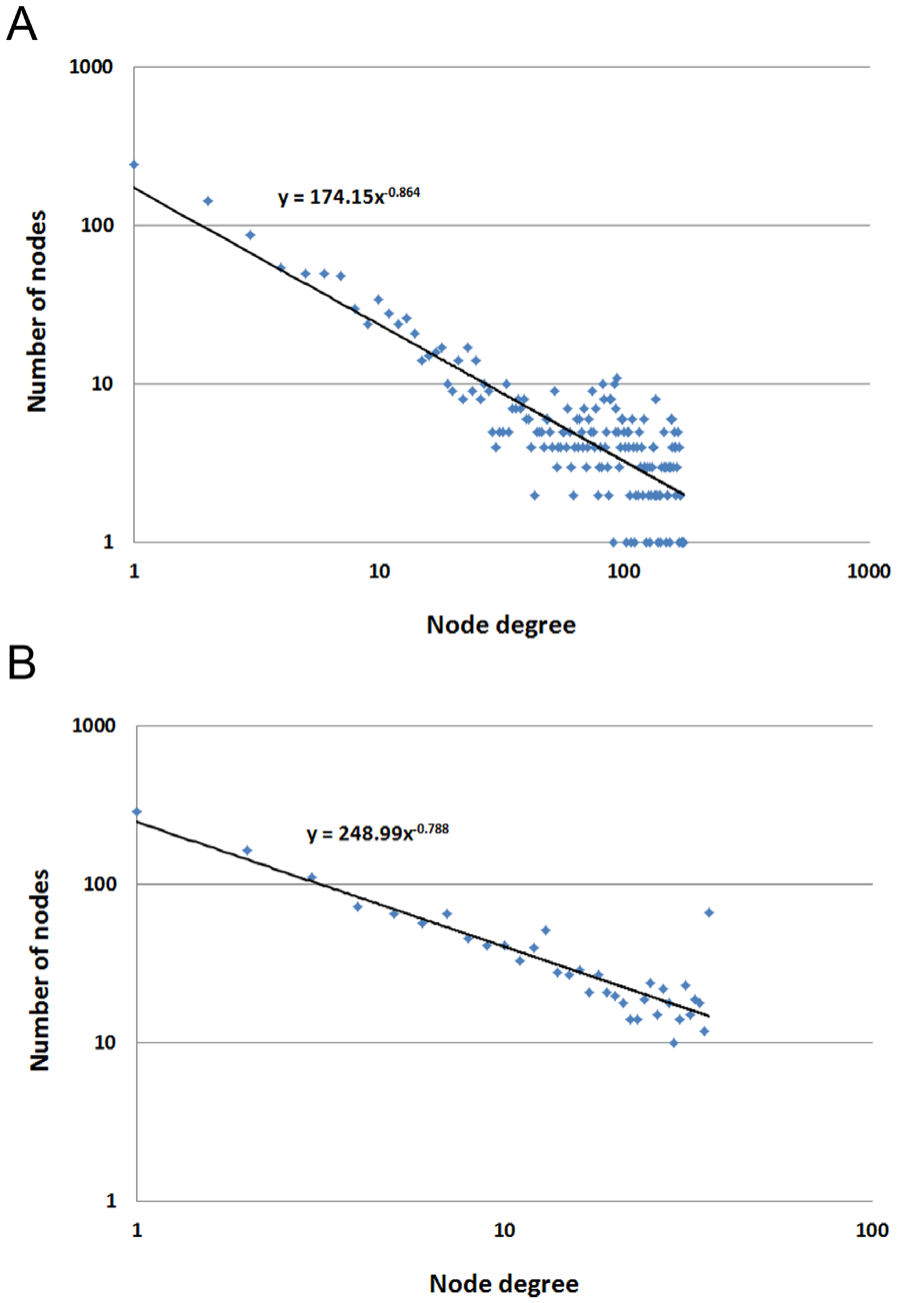
Degree distribution of the drought-responsive gene co-expression network. A, the preliminary network without the maximum connection. B, the final drought-responsive gene network.

The preliminary network was constructed by connecting genes that had an *r*-value>0.71; the network contained 1,618 nodes and 29,688 edges. As would be expected for a biological network, it displayed scale-free topology, whose degree distribution followed a power law with the exponent (Figure 2A). The average degree of the drought-responsive network was 36 at the correlation coefficient cutoff of 0.71. We therefore set the maximum link limit to 36 for each node to avoid some genes in the highly intercorrelated module may have many links to genes in other functional modules. According to the connection threshold, edges connecting two genes in each other's top 36 correlation were retained and other links were removed from the preliminary network. The resulting drought-responsive gene co-expression network consisted of 1,573 gene nodes and 8,730 edges with the average correlation coefficient of 0.83. The degree distribution showed the final network also followed a power-law distribution (Figure 2B).

### Detection of functional modules

**Figure 3:**
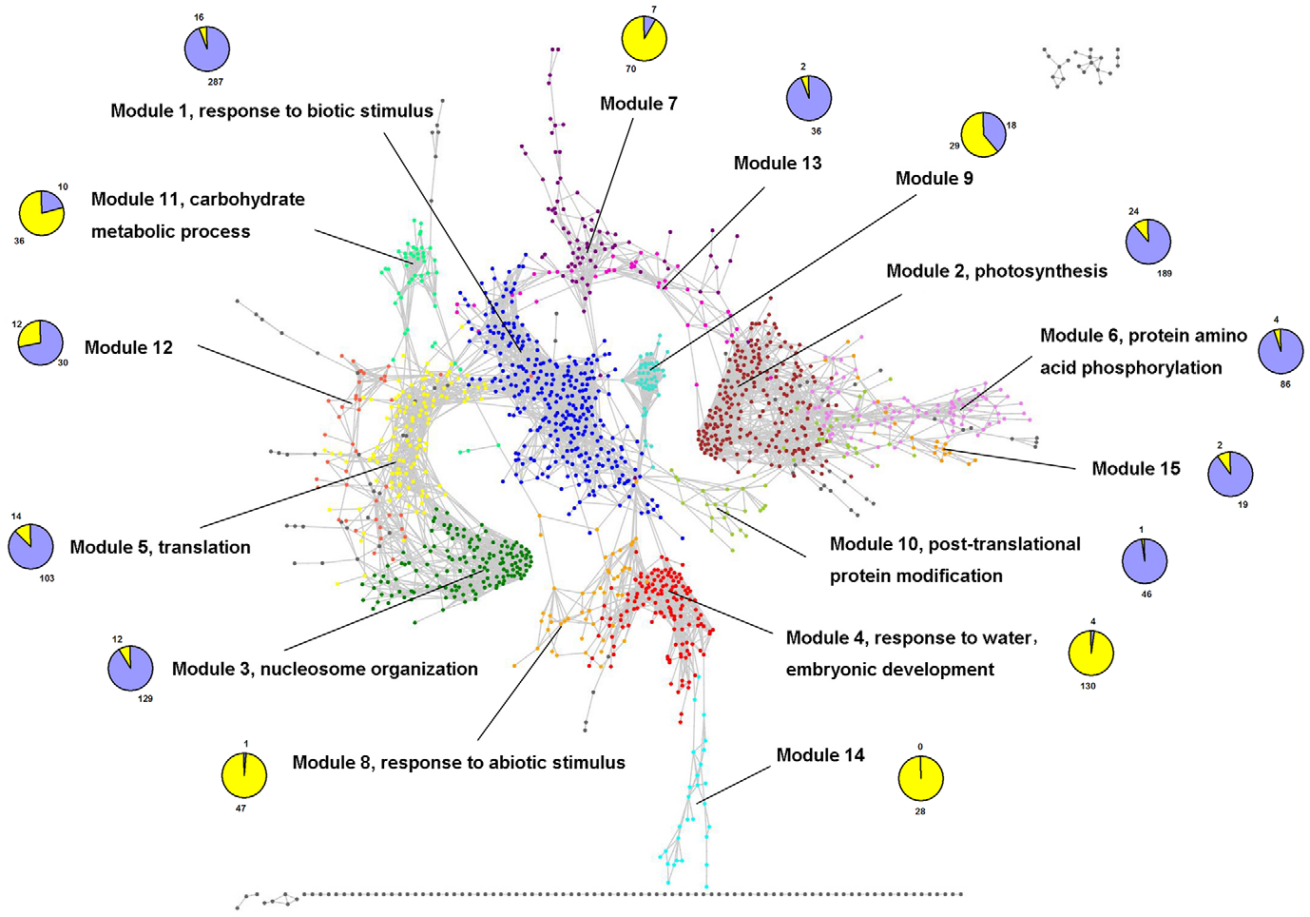
Mapping the modules onto the drought-responsive gene co-expression network. The nodes are color-coded by modules and gray nodes represent genes unassigned to a module. The over-represented GO terms are shown for each module. Each pie chart represents the proportion of up- (yellow color) and downregulated (blue color) genes in the corresponding module.

Responses to drought stress are usually organized as relatively separable modules of highly interconnected genes in the co-expression networks. Graph-clustering techniques are ideal for partitioning large gene networks into biologically significant clusters. Here, we used the Markov Cluster (MCL) algorithm [16], and identified 15 functional modules ranging in size from 21 to 303 genes in the drought-responsive network (Table S2). All 15 drought-responsive modules involved 1,392 highly interconnected genes, which represented more than half of the differentially expressed genes identified from five microarray datasets. These gene modules mapping onto the drought-responsive gene correlation network are shown in Figure 3, and the full data is available as a Cytoscape session file of Dataset S1.

**Figure 4:**
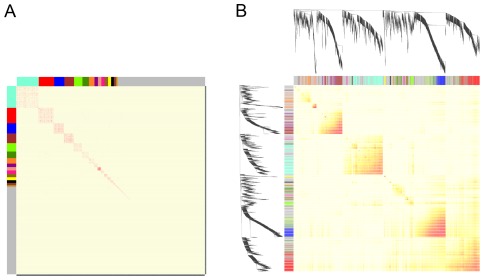
Heat map derived from the drought-responsive gene co-expression network. A, heat map of our network. Genes in the rows and columns have been ordered by a MCL algorithm. Each of the colored bars along the top horizontal and left vertical axes represents a gene module. Genes that do not belong to any module are colored gray. B, heat map of the WGCNA network based on power (β = 4). The genes are colored by module assignment in A.

To determine whether these gene modules in our drought-responsive gene network were robust, another gene correlation network was generated using the WGCNA package [17]. By using this method, a total of 16 modules of highly correlated genes were detected in the WGCNA correlation network. Although the number of gene modules of two networks is not equal, module assignment of our network was highly preserved in the WGCNA network (Figure 4). These data indicated that our method was robust for the construction of gene correlation networks and module detection.

### Modules related to biological functions

One of the aims of network analysis is to identify sets of functionally related genes based on the high gene connectivity in expression. To determine the drought-responsive modules comprised of functionally similar genes, we carried out gene ontology (GO) enrichment analyses for all modules [18]. Functional enrichment revealed that these drought-responsive module genes were preferentially involved in the biological processes of stimulus response, photosynthesis, nucleosome assembly, embryonic development, translation and protein amino acid phosphorylation (Figure 4; a full list is provided in Table S3).

Module 1 consists of 303 gene nodes and is the largest module in the drought-responsive gene co-expression network. It is not surprising that the module is enriched with stimulus response genes (GO: 0050896, FDR = 2.1×10^−11^). There are a total of 119 genes whose functions are associated with responses to environmental stimuli, which represent approximately half of the annotated genes in the module. Most genes in the module are downregulated by drought stress, while only 16 genes are induced by dehydration, which implies that the module may serve as a negative regulator in rice response to drought stress.

Another important negative player is module 2, which contains 213 drought-responsive genes; 189 genes are downregulated by drought stress. Functional enrichment shows that the module genes are enriched with products that target the thylakoid (GO: 0009579, FDR = 1.9×10^−14^) and participate in photosynthesis (GO: 0015979, FDR = 1.3×10^−5^).

Module 4 is enriched for genes known to control the response of plants to water (GO: 0009415, FDR = 3.7×10^−8^). The module contains five genes involved in this process, which accounted for 41.6% of the annotated water responsive genes in the background datasets. Functional enrichment revealed that the module was also enriched for genes involved in embryonic development (GO: 0009790, FDR = 4.7×10^−6^), including six annotated late embryogenesis abundant *LEA* genes. It is of interest that almost all genes within the module are significantly induced by drought stress.

Module 8 is another module that is upregulated by drought stress, which is principally involved in the response to abiotic stimuli. The gene module contains 14 abiotic stimulus responsive genes (GO: 0009628, FDR = 1.9×10^−5^), representing approximately 38% of all of the 37 annotated genes in the module. These data suggest that modules 4 and 8 may serve as key positive players in rice response to drought stress.

Modules 6 and 10 are other two important gene groups that are preferentially involved in the processes of protein amino acid phosphorylation (GO: 0006468, FDR = 7.1×10^−12^ for module 6, and FDR = 3.8×10^−3^ for module 10, respectively). There are 39 annotated genes that encode protein kinases, which represent 29% of the annotated genes in the two modules. This implies that these two gene modules may play important roles in the signal transduction networks that are activated in the drought response in rice.

### Tissue-specific expression in modules

**Figure 5:**
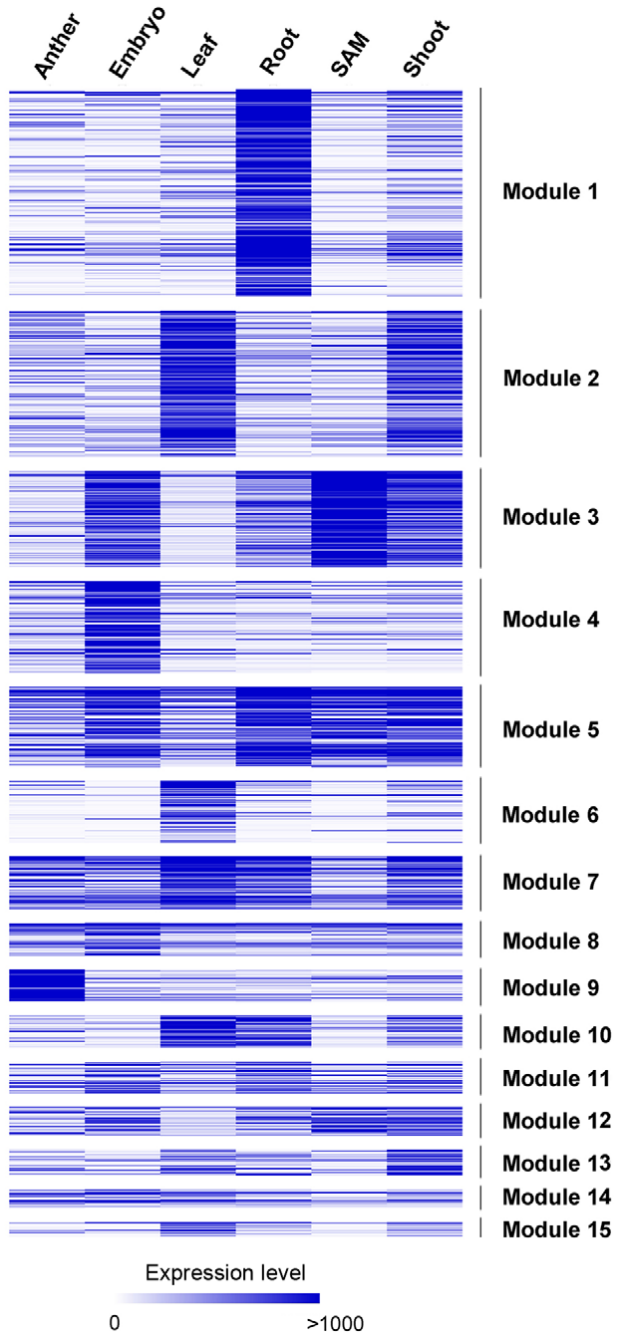
Tissue-specific expression of genes in the drought-responsive modules. The average expression level for each gene in different tissue types was calculated based on the normalized Affymetrix array data.

Expression patterns of drought-responsive module genes were investigated in six different tissue types, including the anther, embryo, root, leaf, shoot and shoot apical meristem (SAM), which are shown in Figure 5. As the largest gene group in the drought-responsive network, module 1 is enriched for stimulus response genes with high expression levels in roots. Transcripts of module 2 are abundant in leaves and shoots, which is consistent with the fact that the module genes are preferentially involved in photosynthesis. Not surprisingly, module 4, which is enriched for *LEA* genes, is highly expressed in the embryo. It should be noted that genes within module 9 show high expression levels in the anther, whereas there is no significant functional enrichment for these genes.

### *Cis*-regulatory elements enriched in modules

**Figure 6:**
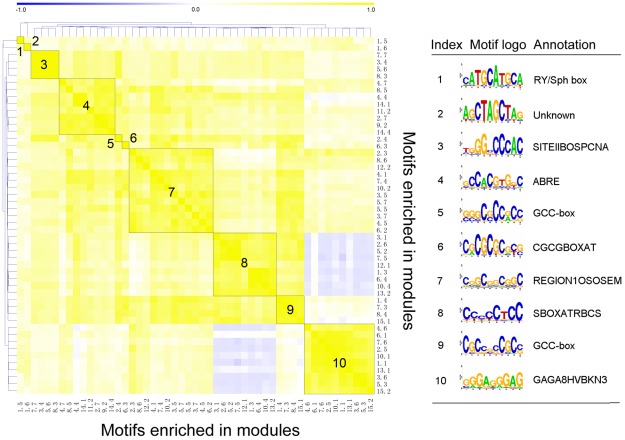
Heat map of the putative *cis*-regulatory motifs enriched in the drought-responsive modules. The motifs in the rows and columns have been ordered by simple hierarchical clustering. A gradient of colors represent the Pearson correlation coefficients between motifs. The black line rectangles in the heat map indicate the similar motifs with a correlation coefficient >0.65. Functions of the merged motifs are indicated in the right-sided table.

Genes in the drought-responsive modules are likely to possess the same *cis*-regulatory motifs in their promoters. To gain an overall view on motif organization of drought response in rice, the putative binding site motifs enriched in drought-responsive gene modules were investigated by MEME tool [19]. A total of 51 putative *cis*-motifs were found in 15 drought-responsive modules with MEME E-value less than 1. The Pearson correlation coefficient between each motif pair was calculated based on the corresponding position weight matrices and similar motifs were clustered using a correlation coefficient cutoff of 0.65. After merging motifs within each cluster, 10 nonredundant motifs were identified to be involved in the response to drought stress (Figure 6). A comparison of the discovered motifs with the *cis*-regulatory elements from the PLACE and AGRIS databases showed that some motifs, such as ABA-responsive element (ABRE) and GCC-box, were known to be involved in drought stress response [20]. As expected, the ABRE element with the conserved GCCACGTGKC sequence was found to be enriched in six modules, including 4, 8, and 14, which were significantly induced by drought stress. Among those revealed elements, most motifs were described to be involved in known functions, while only one motif with the AGCTAGCTAG sequence had an unknown function. We found many of discovered motifs were enriched in more than one drought-responsive gene module, especially the three global motifs that included the REGION1OSOSEM (CSGCGGCGGC), the SBOXATRBCS (CCCCCTCC) and the GAGA8HVBKN3 (GGGAGGGAG) element which occurred in nine or more modules. Interestingly, the RY/Sph box with the conserved CATGCATGCA sequence and the AGCTAGCTAG element were identified to be uniquely enriched in module 1. Although the role of the two motifs in drought stress response remains unclear, the root-specific expression of associated genes suggests that both of them are likely to be involved in transcriptional repression in rice root in response to drought stress.

### Modules are significantly associated with drought response

The primary goal of this analysis was to identify functional modules in the co-expression network that were significantly associated with drought-stress response in rice. To identify such modules, we used the average value of the absolute log_2_-fold expression changes of module genes to measure module changes in the response to drought stress. The significances of module changes greater than the network average were assessed using t-tests in the drought-tolerant (N22, Azucena and Bala) and drought-sensitive (IR64) rice cultivars based on the independent datasets. The data showed there were one to five modules whose expression changes were significantly greater than the network average in each rice variety (Table 1). It was interesting that module 4 had a significantly greater change in gene expression in all investigated drought-tolerant and drought-sensitive rice varieties. Module 4 consists of 134 genes with an average correlation coefficient of 0.88, and most are induced by drought stress in different rice varieties. These data suggested that module 4 was significantly associated with drought-stress response of rice with different genetic backgrounds.

**Table 1:** Module significance in response to drought stress in different rice varieties.

Rice varieties	Microarray datasets	Modules															Unassigned genes	Whole network
		1	2	3	4	5	6	7	8	9	10	11	12	13	14	15		
IR64	E-MEXP-2401	1.68 (0.089)	1.11 (2.88E-08)	1.75 (0.055)	2.82* (2.20E-16)	1.38 (0.103)	0.54 (3.48E-16)	2.01* (8.17E-04)	2.43* (8.03E-07)	1.07 (0.004)	0.70 (6.04E-07)	0.86 (5.56E-05)	1.20 (0.048)	1.56 (0.994)	2.91* (1.67E-09)	1.03 (0.040)	1.47 (0.315)	1.56
N22	E-MEXP-2401	1.61 (0.0159)	1.11 (3.50E-05)	1.59 (0.155)	2.98* (2.20E-16)	0.88 (2.05E-07)	0.97 (1.50E-04)	0.83 (4.20E-06)	1.87 (0.011)	0.97 (0.005)	1.23 (0.211)	1.72 (0.097)	0.87 (0.001)	1.43 (0.962)	1.42 (0.947)	1.80 (0.150)	1.22 (0.014)	1.44
IR64	GSE6901	1.02 (9.21E-08)	1.55 (0.139)	1.13 (0.007)	2.61* (2.20E-16)	1.02 (7.00E-04)	0.79 (3.36E-06)	2.36* (2.25E-10)	1.19 (0.214)	1.36 (0.746)	0.67 (5.51E-05)	1.50 (0.678)	1.20 (0.264)	1.08 (0.097)	4.89* (2.20E-16)	1.10 (0.256)	1.32 (0.326)	1.42
Azucena	GSE24048	0.61 (1.04E-08)	0.91 (0.223)	0.42 (3.26E-09)	1.51* (3.26E-09)	0.57 (6.46E-05)	1.85* (2.03E-11)	0.95 (0.693)	2.17* (5.92E-11)	0.83 (0.287)	1.67* (1.14E-04)	0.86 (0.398)	0.71 (0.101)	0.59 (0.028)	2.41* (5.39E-10)	1.37 (0.157)	1.26 (0.006)	1.01
Bala	GSE24048	0.76 (1.59E-05)	0.95 (0.156)	0.44 (8.44E-10)	1.70* (5.12E-08)	0.53 (1.36E-06)	1.87* (9.88E-10)	0.99 (0.543)	2.15* (4.22E-09)	1.18 (0.547)	1.70* (4.69E-04)	0.76 (0.078)	0.68 (0.036)	0.66 (0.037)	2.52 (7.61E-10)	1.58 (0.054)	1.28 (0.031)	1.07

Each column corresponds to module expression change in the drought stress response, row to a rice variety. Each cell contains the average of absolute log2-fold expression changes of module genes and the *p*-value for differences between the module average and the network average calculated by the t-test. The stars indicate that the average changes of modules are significantly greater than the network average (*p*<0.001).

**Figure 7:**
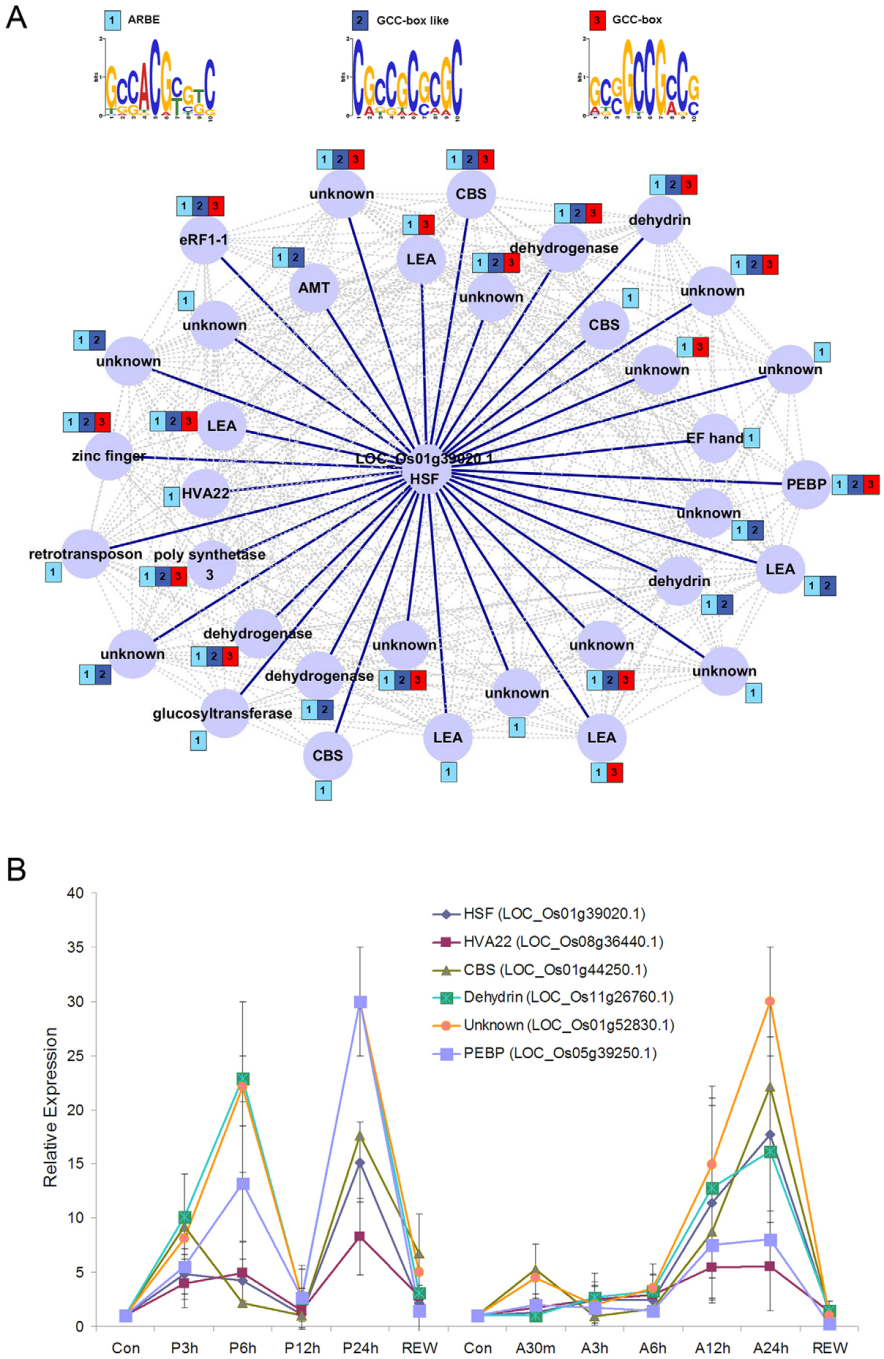
Co-expression relationship of *HSF* with its related genes. A, gene co-expression network and putative binding sites for HSF. B, the average expression levels of *HSF* and its neighborhood genes under PEG and ABA treatments. The error bars represent standard deviation from the mean.

Module 4 contains four genes encoding transcription factors from three families. Interestingly, a heat shock transcription factor (HSF, LOC_Os01g39020.1) induced by drought stress had the highest intramodular connectivity value in the drought-responsive TFs. The *HSF* gene contained 35 co-expression neighbors and their average correlation to the locus was 0.90 (Figure 7A). Many *HSF* co-expression genes encode stress-related functional proteins, such as the LEA and dehydrin proteins [21]. Prediction of *cis*-regulatory elements revealed that the ABRE element and the GCC-box were conserved in these putative target genes; in particular, the ABRE element was found in all 35 neighboring genes. To test the reliability of the inferred HSF regulatory network in the response to drought stress, the *HSF* and its five neighboring genes were further investigated for their stress response by quantitative RT-PCR analysis in the upland rice cultivar, IRAT109. Expression analysis showed that these genes, including *HSF*, were induced by drought stress (simulated by polyethylene glycol, PEG) and abscisic acid (ABA); furthermore, their expression patterns exhibited obvious coherence in IRAT109 seedlings under PEG and ABA treatments (Figure 7B). These results suggest that the ABRE element is probably recognized by the HSF and these co-expression genes may be involved in the ABA-dependent drought response pathway in different rice varieties.

### Organization of hub genes on rice chromosomes

The highly connected hub genes within the networks are thought to play important roles in organizing the functional modules. Here, we identified hub genes as the top 20% highly connected nodes in each module, and 262 hub genes were found in fifteen drought-responsive modules. Functional enrichment revealed that these drought-responsive hub genes were preferentially involved in the response to stimulus (Table 2), which was in accordance with the important roles of these genes in response to drought stress.

**Table 2:** Functional enrichment of drought-responsive hub genes.

GO term	Ontology	Description	FDR
GO:0050896	P	response to stimulus	0.00031
GO:0006950	P	response to stress	0.00079
GO:0009628	P	response to abiotic stimulus	0.001
GO:0015979	P	photosynthesis	0.0011
GO:0009579	C	thylakoid	8.70E-09
GO:0009536	C	plastid	3.50E-06
GO:0005737	C	cytoplasm	0.0076

**Figure 8:**
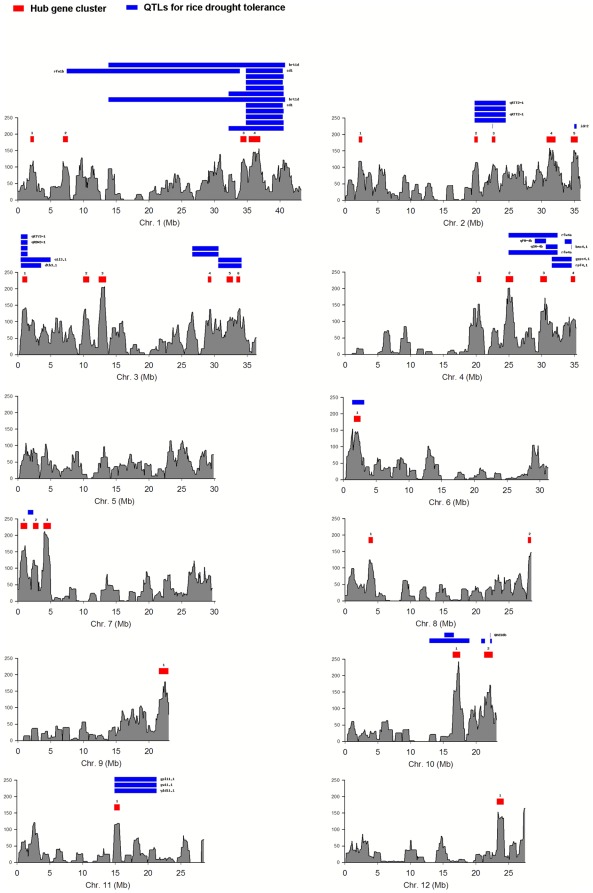
Distribution of hub gene clusters and the overlapped drought tolerance QTLs in rice chromosomes. Red and blue filled rectangles represent hub gene clusters and QTLs for rice drought tolerance, respectively.

Although some data have shown that co-regulated stress response genes tend to cluster in rice chromosomes [22], [23], the connectivity organization of these drought-responsive genes in chromosomes is still unclear. As the intramodular connectivity was more strongly correlated with functional significance than the whole network connectivity, we analyzed the distribution of gene intramodular connectivity values to reveal the organization of hub genes on rice chromosomes. Figure 8 shows the distribution of gene intramodular connectivity values in 1-Mb intervals on each rice chromosome. The distribution of hub genes varied both among the chromosomes and specific regions within each chromosome. Chromosomes 1, 2, 3 and 4 contained more hub genes, while only a few hub genes were found in chromosomes 5, 6, 11 and 12. In several chromosomal regions, relatively larger values of intramodular connectivity were mapped as hub gene clusters (intramodular connectivity value >100; corresponding chromosomal region longer than 0.5 Mb). A total of 30 hub gene clusters were found in the rice chromosomes.

Compared to the mapping of quantitative trait loci (QTLs) for drought tolerance on rice chromosomes (QTL data from Q-TARO database, http://qtaro.abr.affrc.go.jp/) [24], we observed a significant association between the hub gene clusters and QTLs for drought tolerance, and a total of 18 hub gene clusters overlapped with these QTLs, which was far beyond expected by chance (*p*<0.005) (Figure 8). For instance, the hub gene cluster of chr10.1 overlapped with two QTLs that were associated with the root penetration index and the drought response index [25], [26]. It is interesting that this hub gene cluster contains nine drought-responsive genes and three of them (LOC_Os10g31660.1, LOC_Os10g31680.1 and LOC_Os10g31720.1) encode glycine-rich cell wall structural proteins in module 1. It is possible that the reduction of glycine-rich proteins is associated with the remodeling of vessel cell walls in rice roots during drought stress [27]. These data suggest that hub gene clusters are likely to be major components of the QTLs for drought tolerance in rice.

## Discussion

Most of the existing studies used the Pearson-based correlation method to construct co-expression networks, but value-based methods are significantly limited by a homogeneous correlation threshold for all genes in the network [28]. In the study, we propose an integrative approach to construct gene co-expression networks. The preliminary gene network was constructed with the relative stringent correlation threshold in order to reduce false connections of weak correlations between drought-responsive genes. In order to avoid some genes in the highly intercorrelated module that may have many links to genes in other functional modules, we limited the maximum link number for each node, and any edges connecting two genes that did not belong to top 36 reciprocal ranks were removed from the network. Network module analysis showed module assignment of our network was highly preserved in the WGCNA network, and it meant our method was a robust model to detect functional modules in gene correlation network.

The major objective for this study was to use the network-based approach to identify drought-responsive gene modules, providing new insights into the organization of functional modules in the response to drought stress. From publically available microarray data, we identified 2,607 rice genes that showed significant changes in gene expression under drought stress and more than half of the drought-responsive genes were highly intercorrelated to form 15 functional modules. These correlated transcriptional modules are biologically plausible, with enrichments for genes in common functional categories, stress response changes, tissue-specific expression and TF binding sites. Functional annotations show that some modules are enriched for genes involved in the response to stress, including *OsbZip23*, *OsbZip72* and *AP37* which have been validated as important factors affecting drought tolerance traits for rice [29]–[31]. Although approximately 20% of module genes still have not been functionally annotated, the high degree of transcriptional connectivity allows us to infer the role of these novel genes in drought response based on the known annotations of the stress-related genes in the modules.

Among these identified drought-responsive modules, module 4 consisting of 134 genes was significantly associated with drought response in both drought-tolerant and drought-sensitive rice varieties. The involvement of many of these genes in drought response was not previously known, but it was understood that they were involved in controlling the response of the plant to water and embryonic development. Motif enrichment analysis revealed approximately 70% genes in the module contained the ABRE element in their promoter regions, and most of them are induced by drought stress [32], [33]. The enrichment of ABRE element in module 4 suggests that the module may be involved in ABA-mediated drought response pathway [1], [20]. The ABRE element has been shown to be recognized by the bZIP belonging to the AREB/ABFs family [34]–[37], but interestingly, but interestingly, we found a novel candidate gene in module 4 that encodes a heat shock transcription factor which binds to ABRE elements. The HSF TF contains 35 putative target genes with at least one ABRE element in their promoter regions, implying that it is likely to serve as a key regulator of the module involved in drought response.

Gene organization in rice chromosomes is non-random with respect to the complex traits of drought tolerance. The distribution of drought-responsive hub genes that was detected varied both among the rice chromosomes and within regions of each chromosome. Many hub genes in the drought-responsive modules were clustered in rice chromosomes. These hub gene clusters overlapped with QTLs for drought tolerance, which suggests that hub gene clusters are likely to be major components of drought tolerance QTLs. This evidence should facilitate the identification and cloning of drought-related genes at target QTLs. The interesting association between hub gene clusters and drought tolerance QTLs might be the key to understanding the complex genetic basis of drought tolerance in rice.

## Materials and Methods

### Expression data

The dataset of 852 Affymetrix rice arrays was downloaded from the NCBI Gene Expression Omnibus (platform accession number, GPL2025) and the EBI ArrayExpress Archive. Thirty-seven slides were removed from the dataset due to genomic DNA hybridization. A total of 815 slides remained and these slides were normalized using the RMAExpress software for network construction [38].

### Mapping of probe sets to rice loci

The Affymetrix rice genome array was designed based on the early annotation of rice genome, and not all probe sets had one-to-one mapping to rice genes. Some probe sets matched more than one gene, and some were redundant with multiple probe sets mapping to a single gene. To avoid such ambiguous information, we mapped the probe sets to gene models by searching the newly released genome data (version 6.1) of the MSU Rice Genome Annotation Project [39]. The Affymetrix array consists of 57,381 probe sets, each consisting of 11 probes. Mapping of probes required at least seven of the 11 probes within a probe set to match a gene model exactly. According the mapping threshold, 42,059 probe sets were successfully mapped to 47,297 rice genes. We removed 8,549 probe sets that matched multiple genes, as well as 7,773 probe sets that were redundantly mapped to a single gene. After filtration, the retained 25,737 probe sets all had one-to-one mapping to rice genes. The corresponding genes of these unique probe sets were analyzed for the identification of differentially expressed genes.

### Identifying differentially expressed genes

Identification of the drought-responsive genes was based on the normalized expression profiling data of rice seedlings (NCBI GEO: GSE6901), and the Azucena and Bala rice varieties (NCBI GEO: GSE24048), as well as N22 and IR64 (EBI ArrayExpress: E-MEXP-2401) under drought stress conditions. Average signal intensities of biological replicates for each sample were used to calculate the fold change of gene expression. The differentially expressed genes were identified using significance analysis by t-tests with *p*<0.05 and at least two-fold changes (either up- or downregulation). To reduce the noise within the gene co-expression network, the data subsets were restricted to genes that were differentially expressed in at least two out of the five experimental sets.

### Threshold selection and network construction

The correlations between genes were determined by the Pearson correlation coefficient, and it was calculated using Perl script based on the following formula:
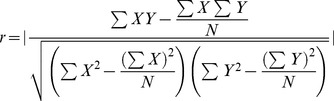
where r is the absolute Pearson correlation coefficient, X and Y represent the corresponding expression profiles of two genes, and N denotes the number of data points in each expression profile.

The density of a gene co-expression network D was defined as a ratio of the actual number of links to all possible links of the non-singleton nodes. It uses the following formula:
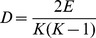
Where E was the number of actual links and K(K−1)/2 was the number of possible links of non-singleton nodes (nodes that were connected to at least one other node).

To determine a biologically relevant *r* cutoff, we apply the approach as described by Aoki et al. to examine the changes in the gene correlation network density as a function of *r* cutoff values [15]. We found that the network density decreased as the cutoff value increased, but the network density was shown to increase as the cutoff was greater than 0.71. According to the relationship between network density and the correlation coefficient, we chose 0.71 as the cutoff in this study.

The value-based co-expression network was created for the drought-responsive genes. An edge in the network represented two genes with a correlation coefficient greater than 0.71. We then limited the maximum link number for each node to reduce the connections between genes in different modules. The edges connecting two genes in each other's top correlation were kept and other links were removed in the network. The resulting co-expression network was drawn using Cytoscape [40].

### Module detection and functional enrichment

Gene co-expression networks with thousands of nodes are difficult to visualize and analyze. A useful strategy for analyzing such a network is to partition it into sub-networks as modules that share regulatory mechanisms and functional relationships. Many graph-clustering algorithms have been developed for partitioning graphs into node clusters based on structure topology [16]. The MCL algorithm as an efficient graph clustering method has been successfully applied to detect modules in gene networks. In this study, we used the MCL method to detect the functional modules in the drought-responsive network with an inflation value of 1.2 (the command: mcl RiceDRNet –I 1.2 –abc).

The drought-responsive modules were tested for GO annotation enrichment using the agriGO program [41]. The statistical significance of the functional enrichment within gene modules was evaluated using the hypergeometric distribution adjusted by the Bonferroni correction for the testing of multiple hypotheses. A GO term was significantly enriched in a module if the adjusted *p*-value was less than 0.01 in comparing with the dataset of all MSU rice loci.

### Cis-regulatory element analysis

The sequences of the promoters (1 kb upstream sequences from transcription start site) were extracted from the MSU rice genome database. The upstream sequence groups of co-expressed module genes were analyzed by the MEME program to find *cis*-motifs of between six and ten nucleotides [19]. The motifs that consisted of mono-nucleotide repeat sequences were removed from the analysis. The Pearson correlation coefficient between each motif pair was calculated by CompareACE based on the corresponding position weight matrices in the given DNA strands [42]. Similar motifs were clustered using a correlation coefficient cutoff of 0.65 and each motif cluster was then merged to nonredundant one. The unique motifs were compared with the known regulatory elements from the PLACE and AGRIS databases based on profile–profile alignment for function annotation [43], [44].

### Expression profiling by quantitative RT-PCR analysis

For transcript level analysis of the inferred co-expression genes under stress, IRAT109 plants were planted in plastic trays walled with sandy soil and placed in the greenhouse. Four-leaf-old seedlings were prepared for PEG and ABA treatments. Seedling roots were submerged in 20% PEG solution for drought stress, and seedling leaves were sampled at 0, 3, 6, 12 and 24 h after the treatment, as well as rewatering after 24 h. For ABA treatment, seedling leaves were sprayed with 0.1 mM ABA solution and sampled at 0, 30 min, 3, 6, 12 and 24 h after treatment as well as rewatering after 24 h.

Total RNAs of the collected samples were extracted using TRIzol reagent (Invitrogen). Then, 2 µl RNA sample was reverse-transcribed to first-strand cDNA with AMV Reverse Transcription System (Promega, USA) using oligo(dT) primers. Real-time RT-PCR using SYBR Green I technology on ABI PRISM 7000 Sequence Detection System (Applied Biosystems, Foster City, USA) was performed. A PCR master mixture of 20 µl contained 1×SYBR® Green PCR Master Mix, 1 µl cDNA, and 0.5 µl 10 µM sense and anti-sense primers (see Table S4). The expression level of rice *Actin1* gene (accession number, X16280) was used as the internal control with primers.

### Randomization test

Permutation tests were used to determine whether the overlap between hub gene clusters and drought tolerance QTLs was higher than expected by chance. For the test, we randomly shuffled gene connectivity and chromosome position, and then counted the number (n) of times that the number of random hub gene clusters (intramodular connectivity value >100; corresponding chromosomal region longer than 0.5 Mb) overlapped with the drought tolerance QTLs was higher than the actual overlaps. We repeated this procedure 1,000 times, and the *p*-value was thus generated equal to n/1000.

## Supporting Information

Table S1 **Microarray samples used in the network construction.**
(XLS)

Table S2 **Gene modules detected by MCL in the drought responsive network.**
(XLS)

Table S3 **Functional enrichment of drought-responsive module genes.**
(XLS)

Table S4 **Real-time PCR primers specific for** ***HSF*** **and its neighborhood genes.**
(XLS)

Dataset S1 **Cytoscape session file of the drought-responsive network with the mapping modules.**
(RAR)
